# Working Hours, Sleep Disturbance and Self-Assessed Health in Men and Women: A Multilevel Analysis of 30 Countries in Europe

**DOI:** 10.3389/fpubh.2022.818359

**Published:** 2022-04-05

**Authors:** Aziz Mensah, Susanna Toivanen, Martin Diewald

**Affiliations:** ^1^Bielefeld Graduate School in History and Sociology, Bielefeld University, Bielefeld, Germany; ^2^School of Health, Care, and Social Welfare, Mälardalen University, Västerås, Sweden

**Keywords:** work hours, self-assessed health, sleep disturbance, welfare policies, gender, working adults, moderating role, Europe

## Abstract

**Objectives:**

This study examined the gender and cross-country differences in the relationship between working hours and self-assessed health among working men and women in Europe, and further explored the moderating role of sleep disturbance in the relationship.

**Methods:**

We used cross-sectional data from the 6th European Working Condition Survey on 14,603 men and 15,486 women across 30 countries in Europe. A multivariate logistic regression was applied to evaluate the relationship between working hours, sleep disturbance, and self- assessed health. In addition, we employed a two-stage multilevel logistic regression to assess the cross-country variations in the relationship between working hours and self-assessed health.

**Results:**

The study showed a slightly U-shaped relationship between working hours and less-than-good self-assessed health among working adults in Europe (<31 h: aOR = 1.11; 95% CI: 1.00–1.25, 41–50 h: aOR = 0.98; 95% CI: 0.84–1.15, and 50+ h: aOR = 1.31; 95% CI: 1.07–1.59). However, working men had higher odds of reporting less-than-good self-assessed health as compared to women when they devote longer hours to paid work. The results further showed that there are cross-country variations in the association between working hours and less-than-good self-assessed health for both men and women, and that men had slightly lower variations as compared to women. Contrary to expectation, sleep disturbance did not moderate the relationship between working hours and less-than-good self-assessed health for both men and women in Europe.

**Conclusions:**

Although there are gender differences and cross-country variations in the association between working hours and less-than-good self-assessed health, sleep disturbance did not moderate the associations. These findings underscore the importance for strict work time regulation and generous work-family policies that may promote good working conditions and health.

## Introduction

International time use data indicate that average weekly hours spent in paid work reduced from 59.5 h in 1900 to 45.40 h in 1950, and subsequently to 41.70 h and 40.1 h in 1970 and 2000 respectively (1). Similarly, recent studies suggest that weekly hours spent in paid work have subsequently reduced to 40 h in many countries around the world (2). Also, a report conducted by the Organization for Economic Cooperation and Development (OECD) in 2019 indicated that time spent in paid work reduced below 40 h in most countries in Europe, with Netherland, Norway, and Switzerland reporting the lowest working hours respectively (3). Despite these reductions, there are several reports about longer hours in paid work in many countries including United Kingdom, Eastern European countries (4), Israel, Costa Rica, Mexico, Columbia, Turkey (5), China, Korea, Japan (2). This may be due to increased workload, tight deadlines, organizational commitment, recognition, promotion, and job insecurity that many workers experience (6).

Although longer hours in paid work may be beneficial to financial and economic stability, physical health, and well-being (7), this correlation may not be linear. Rather, longer working hours may become detrimental to health due to stress and work-life conflicts (8–10). Several studies have suggested that longer hours in paid work may be linked with poor physical and mental health outcomes such as coronary heart disease, stroke, diabetes, depressive symptoms, and anxiety (8–15). Other studies have found significant relationship between longer hours in paid work and health-related behaviors such as sleep disturbance (16). However, there has been mixed results as some studies have observed no association between long working hours and adverse health outcomes (15, 17) which may be attributed to societal expectation of gender roles, and work and family friendly policies that exist in the country (18).

There is still a gender difference in time spent in paid work. While women have increased hours in paid work over the years, men still allocate more time to paid work (4, 19). Even when women catch-up with working full-time, men do significantly more overtime work than women (18). However, it is important to note that women continue to spend more time in unpaid work in terms of care responsibilities and housework activities than men (20). Even when women and men spend the same amount of time in paid work, there is still an imbalance in the amount of time that women spend in unpaid work as compared to men. Crompton (19) attributed the gender difference in paid work to the societal expectation and gender norms. Countries with breadwinner/female care giver model expect men to engage in paid work while women are expected to engage mainly in unpaid work. On the other hand, countries with dual-earner/dual-caregiver model encourage both men and women to engage in paid and unpaid work. Due to the societal expectation of gender norms, prior studies have found gender difference in the association between paid work hours and negative health outcomes (18, 21), and others found inconsistent (18, 21) or similar associations between women and men (22). Contrarily, a recent systematic review and meta-analysis found that there is no gender difference between long working hours and occupational health problems (23).

Regarding cross-country differences in paid work, both Esping-Andersen (24) and Gornick and Meyers (25) have put forward a few contributing factors. Firstly, countries with generous parental leave policies and affordable and quality child care allow mothers with young children to take time off from paid work for conducting unpaid work without exiting the labor market (25). According to Gornick and Meyers (25), Nordic countries with more generous parental leave policies of about 30–42 weeks for mothers and comparatively generous benefits for fathers have a higher proportion of the population engaged in paid work as compared to conservative countries with 12–16 weeks of paid parental leave for mothers and generally weak benefits for fathers (25).

Secondly, work time regulations in European countries may contribute to the difference in time spent in paid work. Although the European Union (EU) work time directive has limited the number of hours spent in paid work to 48 h in a week (26), some counties in Europe, particularly the Nordic and Conservative countries have set their normal work time to 35–39 h in a week (25). Nevertheless, Plantega and Remery (26) found evidence that many countries have deviated from the EU work time directive by taking advantage of the framework of the collective agreement and the flexibility in the directive in relation to sectors and type of job (26).

Thirdly, national difference in labor laws and employment relations, particularly in relation to collective bargaining, minimum wage, trade union density, and employment protection laws may also influence the difference in time spent in paid work among workers in Europe. While most countries in Europe use minimum wages as a mechanism to help prevent unfair pay and compensation, countries like Italy, Austria, Switzerland, and Cyprus do not have a minimum wage (27). In these countries, wages are normally negotiated by unions and employment associations in terms of collective agreement which regulates the lowest pay in the labor market. However, some companies in these countries do not have a collective agreement for pay and working conditions, and employers may decide the hourly payment of their workers, which may sometimes be unfair. Thus, there is a possibility that workers may be forced to prolong working hours to meet the pressing financial demands of their families.

Previous research on cross-country comparison has indicated that the differences in welfare policies may influence the association between paid working hours and self-assessed health (SAH) (8, 28). For instance, a study conducted by Artazcoz and colleagues (28) demonstrated that countries with less generous welfare policies have a higher magnitude of association between long working hours of paid work and poor health outcomes, particularly among women. Similar gender patterns were found in countries with more generous welfare policies although long working hours of paid work were less likely to be related to adverse health outcomes.

Biological function in terms of sleep is needed to maintain good physiological and psychological health (29). Numerous studies have shown gender difference in sleep hours (30, 31), where women experience longer sleep hours than men (32). Nevertheless, other studies have suggested that even though women sleep longer than men, women experience more disruptive and poorer sleep quality than men (32, 33). It is well established that adults need about 7 h of sleep on a daily basis for best survival (34). Thus, due to its general relevance for health, sleep disturbance may be a candidate mechanism for explaining how long working hours are related to perceived health.

Time spent in paid work may affect sleep in various ways, which may consequently result in adverse health outcomes (35). Working more than 55 h a week was significantly associated with shorter sleep hours or sleep disturbance than working 35–40 h a week (16). At the same time sleep disturbance may also be related to adverse health outcomes such as poor SAH, mortality and morbidity, obesity, and back pain (36–39). Thus, it is plausible that the relationship between long working hours and SAH may be modified by sleep disturbance (38).

Previous studies on the relationship between working hours and SAH in working adults have mainly focussed on a single country and gender differences (18, 38). To date, only few studies have focused on a group of countries for international comparison (8, 28). Thus, the present study is among the first to analyse a comprehensive cross-country sample of 30 countries in Europe. Moreover, there is a lack of studies about the moderating role of sleep disturbance for the relationship between working hours and SAH among workers in Europe. The present study investigated the gender-specific interactive effect and cross-country variations in the association between working hours and SAH among workers in Europe. Furthermore, we examined the moderating role of sleep disturbance in the relationship between working hours and SAH among working men and women. Therefore, the following research questions were stated:

1. Are there associations between working hours and less-than-good SAH in Europe? And do these associations differ between men and women?
2. Does the association between hours of paid work and less-than-good SAH vary between countries in Europe and among men and women?
3. Are the associations between hours of paid work and less-than-good SAH moderated by sleep disturbance among men and women?

## Methods

### Data

We used data from the 6th wave European Working Condition Survey 2015 which was conducted by the European Foundation for the Improvement of Living and Working Conditions (Eurofound). The 6th wave EWCS 2015 is a cross-sectional study which covered about 35 countries including the 27 European Union (EU) member states, Switzerland, Norway, United Kingdom (UK), and 5 potential EU candidates (Turkey, Serbia, Albania, Montenegro, and Former Yugoslav Republic of Macedonia). The survey targeted about 44,000 working men and women who were between the ages of 15 years and above. Although, there was a target reference sample size of 1,000 individuals for each country, countries with larger population size like Germany, Italy, France, Spain, UK, Turkey, and Poland were given the opportunity to increase their sample size. Based on a multistage, stratified random sampling method, the target population was selected for a face-to-face interview. Details of sample sizes for all countries, sample design, and sampling principles are provided elsewhere (40).

Since the present study is based on working adults, we limited the analyses to participants who are between the ages of 16–64 years, not disabled, and are engaged in paid work. Furthermore, we restricted our analysis to 30 countries in the European Economic Area including the European Union (EU) 27 countries, 2 countries from the European Free trade Association (Switzerland and Norway), and a former member of the EU (United Kingdom).

### Variables

#### Dependent Variable

SAH was used as the outcome variable. It is well established that this indicator may be used as a good proxy in appraising individual health status, and it is strongly associated with mortality (41). The respondents were asked: “In general, how would you rate your health today”? Responses ranged from 1 (very good), 2 (good), 3 (fair), 4 (bad), and 5 (very bad). In order to compare our results to similar studies in this field of research, and due to the small number of cases for “bad” and very “bad” responses, we dichotomized the SAH where very good and good were considered as “very good or good SAH”, and the remaining three levels were considered as “less-than-good SAH” (42–45).

#### Independent Variables

Working hours, sleep disturbance, and gender were used as the three independent variables for this study. Regarding working hours, participants were asked: “How many hours do you usually work per week in your main paid job”? We categorize working hours into four groups (<31 h, 31–40 h, 41–50 h, 50 + h) in order to compare our findings with previous studies (8, 18, 28). This categorization also helped us to identify part-time work as well as overtime work.

Sleep disturbance was measured with questions which seek to find out about sleep problems of working adults in the last 12 months. The EWCS 2015 measured sleep disturbance with 3 items: “Difficulty falling asleep?” “Waking up repeatedly during the sleep?” and “Waking up with a feeling of exhaustion and fatigue?”. Responses were rated on a Likert scale of 1 (daily), 2 (several times a week), 3 (several times a month), 4 (less often), and 5 (never). We re-coded the scale in ascending order so that higher levels of sleep disturbance will indicate higher values and lower levels will indicate lower values. The validity of this scale has been confirmed previously with a sample in a general population (46). The Cronbach α for this present study showed a good internal consistency of reliability of 0.89.

Another independent variable that was used in this present study was gender. Gender was measured as a binary basis (man or woman).

#### Covariates

Demographic variables, socio-economic positions, working conditions, and countries were used as potential covariates in estimating the association between working hours and SAH (18, 45, 47). Demographic variables that were included in the current study was age, household size, marital status (non-married or widowed, married or cohabiting), and living with child. Also, socio-economic variables included education (primary school or less, secondary, post-secondary, and tertiary), and occupation (International Standard Classification of Occupation-08). Furthermore, we used industry (agriculture, manufacturing and industry, service, others), working arrangement (set by company, can choose between fixed schedule, flexible working time, working time is determined by oneself), control (yes or no), shift work (yes or no), and employment type (self-employed, employed) to measure the working characteristics. Finally, the 30 countries that were included in the analysis were Sweden, Finland, Denmark, Norway, Ireland, UK, Germany, Austria, France, Belgium, Switzerland, Netherland, Luxembourg, Croatia, Bulgaria, Estonia, Hungary, Slovenia, Czech Republic, Slovakia, Poland, Romania, Latvia, Lithuania, Italy, Spain, Portugal, Greece, Malta, and Cyprus.

### Statistical Analyses

Means, standard deviations, and proportions of all measured variables were calculated to understand the general distributional characteristics of the variables by gender. After descriptive analyses, several analytical strategies were adopted. First, we employed the chi-square test for variables that are categorical, point biserial for continuous variables, and rank biserial method for ordinal variables to examine the relationship between the dependent variable, independent variables, and the covariates at the bivariate level. These tests were stratified by gender. Additionally, we adopted the Variance Inflation Factor (VIF) to test for multicollinearity of the independent variables and the covariates that were used to fit the regression model. It is recommended that a VIF value of <10 may be considered as evidence of low multicollinearity or non-existence of multicollinearity (48).

Furthermore, we used multivariate logistic regression to estimate the association between working hours and SAH with adjustments for demographic and socio-economic variables, working conditions, and countries. To test for the gender difference in the association between hours of paid work and SAH among workers in Europe, we performed two additional multivariate logistic regressions. The first multivariate logistic regression was adjusted for the covariates and stratified by gender to obtain the effect for men and women separately. Secondly, we used the full sample to estimate another multivariate logistic regression between working hours and SAH including an interaction term (work hours^*^gender) and adjusted for the covariates. We then employed a Wald test to determine the significance of the interaction term.

Due to the multilevel nature of the data, we employed a two-stage multilevel logistic regression with individual workers (level 1) nested within countries (level 2). This allowed us to estimate for the between country variations in the association between working hours and SAH in Europe. Three models were fitted with the multilevel logistic regression. First, we calculated the empty model (model 1) which contains no exposure variables and provides information of the cross-country variability of SAH (random intercept). The second (model 2) includes working hours and provides information on the between country variation of the association between working hours and SAH. Lastly, the third (model 3) includes model 2 and all the covariates that were used in the study. We employed the Median Odds Ratio (MOR) and the Variance Partition Coefficient (VPC) to examine the level of variability between countries. While the MOR calculates the median values of the odds ratio between the country at lowest risk and the country at highest risk when selecting two countries at random (45, 49), the VPC calculates the percentage of the variation that may occur between countries (50). According to Merlo and colleagues (49), if MOR>1, then there is between country variation. Finally, the Bayesian Information Criterion (BIC) and the Akaike Information Criterion (AIC) were used to provide information on the goodness of the model fit. Since the multilevel logistic regression that was used in this analysis only estimated the overall variation for the 30 countries, we conducted additional multivariate logistic regression which was adjusted for covariates and stratified by country and gender. This allowed us to perform a cross-country comparison of the magnitude of the odds between working hours and less-than-good SAH among workers.

Finally, we tested the moderating role of sleep disturbance in the relationship between working hours and less-than-good SAH among men and women in Europe. Here, we introduced another interaction term (working hours^*^sleep disturbance) in the multivariate logistic regression which was adjusted for covariates and stratified by gender. A Wald test was employed to determine the significance of the interaction term. A sensitivity analysis was performed entering working hours as a continuous variable and then multiplying it by sleep disturbance which is an ordinal variable (working hours^*^sleep disturbance) to determine if the results will be different from the earlier results when working hours was categorized. Sample weights were used in all analyses to correct for under and over sampling. Stata V14 (51) was used to perform the statistical analyses.

## Results

### Descriptive Statistics

Table 1 provides detailed information of the sample characteristics of working adults in the 6th wave of the EWCS 2015. Among the 30,089 study participants, 14,603 (48.53%) were men and 15,486 (51.47%) were women. The average age among working men and women was similar (men = 42.79 ± 11.48 vs. Women = 42.88 ± 11.15). Women engaged in less self-employment than men. A higher percentage of working women were engaged in elementary occupation (11.25%), service and sales (27.74%), and clerical support (12.56%) than men. On the other hand, a higher percentage of men than women worked as managers (8.15%), craft and related trades (20.87%), and plant and machine operators and assembly (11.60%). Overall, women reported higher shares of post-secondary and tertiary education than men in Europe. While most men were found in the industry sector (men = 32.55% vs. women = 12.18%), most women worked in the service and sales sector (men = 58.05% vs. women = 76.60%). Women performed more shift work than men, and had their working time set by their company as compared to men. Also, women reported a slightly higher proportion of less-than-good SAH (21.83%) than men (20.14%). At the same time, women reported slightly higher average sleep disturbance (2.62 ± 1.29) as compared to men (2.36 ± 1.25). While a higher proportion of men engaged in 50 + weekly hours of paid work (9.96%) as compared to women (3.73%), a higher proportion of women engaged in <31 weekly hours of paid work (32.74%) than men (17.54%).

**Table 1:** General characteristics of participants of the European Working Condition Survey 2015.

**Variables**	**Men**		**Women**	
	***N*** **=** **14,603**	**% or Mean** **±SD**	***N*** **=** **15,486**	**% or Mean** **±SD**
**Sleep disturbance**		2.36 ± 1.25		2.62 ± 1.29
**SAH**				
Very good or good	12,482	79.86%	12,732	78.17%
Less-than-good	3,147	20.14%	3,556	21.83%
**Age**		42.79 ± 11.48		42.88 ± 11.15
**Household Size**		2.81 ± 1.33		2.81 ± 1.28
**Marital Status**				
Non-married or widowed	5,094	32.59%	5,789	35.54%
Married or cohabiting	10,535	67.41%	10,499	64.46%
**Living with a child**				
No	8,671	55.48%	7,820	48.01%
Yes	6,958	44.52%	8,468	51.99%
**Education**				
Primary school or less	546	3.49%	458	2.81%
Secondary	9,079	58.09%	8,188	50.27%
Post-secondary	1,201	7.68%	1,386	8.51%
Tertiary	4,803	30.73%	6,256	38.41%
**ISCO**				
Armed forces occupation	116	0.74%	11	0.07%
Managers	1,273	8.15%	826	5.07%
Professionals	2,417	15.46%	3,817	23.43%
Technicians and associate	1,916	12.26%	2,050	12.59%
Clerical support workers	948	6.07%	2,046	12.56%
Service and sales	2,179	13.94%	4,519	27.74%
Skilled agricultural, forestry and fish	495	3.17%	228	1.40%
Craft and related trades	3,262	20.87%	544	3.34%
Plant and machine operators and assembly	1,813	11.60%	415	2.55%
Elementary occupation	1,210	7.74%	1,832	11.25%
**NACE**				
Agric	759	4.86%	352	2.16%
Industry	5,088	32.55%	1,984	12.18%
Service and sales	9,072	58.05%	12,477	76.60%
Other	710	4.54%	1,475	9.06%
**Sector**				
Private	11,756	75.22%	10,242	62.88%
Public	2,950	18.88%	4,946	30.37%
Joint private-public	577	3.69%	581	3.57%
NGO	110	0.70%	247	1.52%
Other	236	1.51%	272	1.67%
**Shift work**				
Yes	3,208	20.53%	3,664	22.50%
No	12,421	79.47%	12,624	77.50%
**Weekly hours**				
30 h and below	2,741	17.54%	5,332	32.74%
31–40 h	8,642	55.29%	8,715	53.51%
41–50 h	2,689	17.21%	1,634	10.03%
50+ h	1,557	9.96%	607	3.73%
**Working arrangement**				
Set by company	9,005	57.62%	9,966	61.19%
Can choose between fixed schedule	997	6.38%	1,386	8.51%
Flexible time	2,971	19.01%	3,057	18.77%
Working time is determined by self	2,656	16.99%	1,879	11.54%
**Type of employment**				
Employee	12,968	82.97%	14,631	89.83%
Self-employed	2,661	17.03%	1,657	10.17%
**Control**				
Yes	8,463	54.15%	8,667	53.21%
No	7,166	45.85%	7,621	46.79%

Then, to check for the average weekly working hours among men and women across countries in Europe, we conducted additional descriptive statistics using weekly working hours as a continuous scale. Results from the Appendix Figures 1, 2 showed that Greece had the highest average weekly working hours for working men in Europe, followed by Romania, Croatia, Slovenia, and Czech Republic. On the other hand, working women in Romania reported the highest average weekly working hours followed by Bulgaria, Croatia, Slovakia, and Hungary. Furthermore, working men and women in the Netherlands and Germany reported the lowest average weekly hours in Europe.

### Bivariate Relationships

Appendix Table 1 depicts the bivariate association between the independent variables, covariates, and the dependent variable. The results showed that there is significant association between level of working hours and SAH among men and women. Sleep disturbance was also negatively associated with SAH for both men and women (men: *r* = −0.346 vs. women: *r* = −0.403). We found a significant but negative association between household size and SAH (men: *r* = −0.054 vs. women: *r* = −0.058). Although we found a statistically significant association between type of employment and SAH for working men, the association was not found for women. The significance of the independent variables, covariates, and the dependent variables at the bivariate level sufficiently justifies the inclusion of these variables in the multivariate analysis (Appendix Table A1).

Results for the VIF's are also provided in Appendix Tables 2, 3. The mean VIF for men and women was quite similar (men = 1.44 vs. women = 1.3). The highest VIF for working men and women was 2.16 and 2.01, respectively, and this suggests that there is no multicollinearity between the independent variables and the covariates.

### Association Between Working Hours and SAH

Table 2 shows information on the association between paid work hours and SAH among workers in Europe. After adjusting for demographic and socio-economic variables, working conditions, and countries, we found a significant association between working <31 h a week and less-than-good SAH [aOR = 1.11; 95% CI: (1.00-1.25)] when compared to the reference group of those working 31–40 h a week. Similarly, working 50 + h a week in paid work had higher odds [aOR = 1.31; 95% CI: (1.07–1.59)] of less-than-good SAH when compared to the reference group. However, the relationship between working 41–50 h a week and less-than-good SAH was not significant when compared with the reference group. Therefore, the results indicate a slightly U-shaped relationship between paid work hours and less-than-good SAH among working adults in Europe.

**Table 2:** Association between paid work hours and SAH among workers from the European Working Condition Survey 2015.

**Variables**	**Full sample**
**Paid work**	**aOR (95%CI)**
<31 h	1.11(1.00–1.25)
31–40 h	1.00(ref)
41–50 h	0.98(0.84–1.15)
50+ h	1.31(1.07–1.59)

*CI, 95% Confidence Interval; aOR, Adjusted odds ratio*.

*Odds ratios are controlled for demographic and socio-economic variables, working conditions, and countries*.

### Gender Differences in the Association Between Working Hours and SAH

Estimate from the Wald test of interaction (*p* = 0.0841) indicates that, overall, there was a significant gender difference in the association between working hours and less-than-good SAH among men and women in Europe after adjusting for covariates. Results of the association between working hours and SAH by gender are shown in Table 3. While we observed a significant association between men who spent <31 h a week in paid work and less-than-good SAH [aOR = 1.18; 95% CI: (1.01–1.42)] in Europe when compared to the reference group, the association was not found among women. Furthermore, we found a significant association between working 50 + h a week and less-than-good SAH [aOR= 1.36; 95% CI: (1.06-1.73)] among men but not women when compared to the reference group.

**Table 3:** Relationship between paid work hours and SAH in Europe by gender.

**Variables**	**Men**	**Women**
**Paid work**	**aOR (95%CI)**	**aOR (95%CI)**
<31 h	1.18(1.01–1.42)	1.08(0.93–1.25)
31–40 h	1.00(ref)	1.00(ref)
41–50 h	0.93(0.75–1.15)	1.17(0.92–1.49)
50+ h	1.36(1.06–1.73)	1.15(0.83–1.61)

*CI, Confidence Interval; aOR, Adjusted odds ratio*.

*Odds ratios are controlled for demographic variables, socio-economic variables, and working conditions*.

### Between-Country Variations in the Associations Between Working Hours and SAH

To examine the cross-country variations in the relationship between paid work hours and less-than-good SAH among workers in Europe, we used the multilevel logistic regression to estimate the random intercept, MOR, and VPC. As showed in Table 4, the random effect for model 2 showed that there are between-country variations in the association between working hours and less-than-good SAH in Europe (men: MOR = 1.55 vs. women: MOR = 1.68). Also, model 3 which is the full sample, indicated that there are significant variations in the association between working hours and less-than-good SAH between countries in Europe (men: MOR = 1.63 vs. women: MOR = 1.74). However, comparing model 2 and model 3 showed that the introduction of demographic and socio-economic variables, and working conditions, as covariates did not add any major reduction in the between country variance in SAH for both men and women in Europe. Nonetheless, the information from the AIC and BIC indicated that model 3 provided a better fit than model 1 and 2. Overall, the percentage change of variation in SAH is slightly higher among women than men (men: MOR = 7.41% vs. women: MOR=9.25%). This outcome was further corroborated in the cross-country comparison of the odds between working hours and less-than-good SAH among workers in Europe that was performed in the supplementary analysis in Appendix Figure 3. For instance, while we found a significant association between working <31 h a week and less-than-good SAH among working men in Czech Republic, Denmark, and Switzerland, the statistically significant association for the rest of the countries were not found. In regard to women, the analysis showed a significant association between working <31 h a week and less-than-good SAH in only Belgium, Denmark, and Romania. Furthermore, we found a significant association between working 50+ h and less-than-good SAH among working men in Portugal but not women. Also, there was significant association between working 50+ h and less-than-good SAH among working women in Romania and Belgium but not men. However, it is important to note that the statistically significant association between working 50+ h a week and less-than-good SAH was not found for the rest of the countries in Europe.

**Table 4:** Between country variation of paid work hours and SAH by gender.

**Random effect**	**Model 1**	**Model 2**	**Model 3**
**Men**			
Between country variation	0.5382	0.4595	0.5131
MOR	1.67	1.55	1.63
VPC	Ref	6.0%	7.41%
BIC	16,618.77	15,356.23	13,614.13
AIC	16,603.37	15,356.23	13,491.62
**Women**			
Between country variation	0.5382	0.5408	0.5792
MOR	1.67	1.68	1.74
VPC	Ref	8.1%	9.25%
BIC	16,618.77	16,612.71	14,358.77
AIC	16,603.37	16,589.62	14,481.95

*MOR, Median Odds Ratio; (VPC), Variance Partition Coefficient; Model 1, Empty model; Model 2, model 1 + working hours; Model 3, model 2+demographic, socio-economic, and work characteristics*.

### Moderating Role of Sleep Disturbance

After adjusting for demographic and socio-economic variables, working conditions, and countries, our results from the Wald test performed on the interaction term (working hours^*^sleep disturbance) was not significant (men: *p* = 0.3630 vs. women: *p* = 0.1313). Similarly, a sensitivity analysis performed by maintaining working hours as a continuous variable showed that sleep disturbance did not moderate the relationship between working hours and less-than-good SAH for working men and women (men: *p* = 0.9938 vs. women: *p* = 0.7018). Taken together, the results suggest that sleep disturbance did not play a moderating role in the association between working hours and less-than-good SAH in men and women in Europe.

## Discussion

Using data from a comprehensive country sample of 30 countries, this study found significant gender differences and cross-country variations in the association between working hours and less-than-good SAH among workers in Europe. However, sleep disturbance did not moderate the association between working hours and less-than-good SAH in men or women.

### Paid Work Hours and SAH

Present findings show that both short (<31 h) and long hours (50+ h) of paid work are associated with less-than-good SAH among employees when compared to the reference group of working 31–40 h a week, indicating a slightly U-shaped relationship. Present results are in line with a systematic review on working populations in Europe, US, and Australia that found a U-shaped association between working hours and stroke (9). On the other hand, some studies have concluded that there is rather a graded association between working hours and adverse health outcomes, where the risk of experiencing health problems increases as working hours increase (13). In view of this, we noted that spending longer hours in paid work may have higher odds of less-than-good SAH than shorter hours. The findings may be attributed to several possible reasons. For instance, working longer hours may reduce rest time and time for recovery (52), and this may subsequently link to health problems such as cardiovascular diseases and mortality (53). Another possible reason is that, spending long hours in paid work may increase exposure to high work demands and other hazardous psychosocial and physical risk factors which may increase employees' stress levels and have an adverse effect on their health (54, 55). The association may also be explained by the fact that long working hours may promote unhealthy behavior such as drinking and smoking which are strongly linked with negative health outcomes (13).

The findings regarding the association between short working hours and less-than-good SAH may also be attributed to the precarious working conditions such as part-time work, shift work, and temporary jobs which accompany short working hours and this may be related to poor health outcomes (56). In addition, it is possible that those who work short hours do so because they already have health problems (healthy worker effect) (57) and this suggest a bi-directional relationship between short working hours and SAH (58). However, such analyses are beyond the scope of the present study because of the cross-sectional nature of the data.

### Gender Differences in Paid Work and SAH

It is well established that there are gender differences in the relationship between paid work hours and negative health outcomes among workers, but there are inconsistencies whether the associations are stronger among men than women or vice versa (18, 21). In line with another study (18), the present study demonstrated that the relationship between paid work hours and less-than-good SAH are more pronounced among men than women. For instance, while we found a slightly U-shaped association in the relationship between working hours and less-than-good SAH among men, there was no statistically significant relationship between paid work hours and less-than-good SAH among women. This particular finding is striking considering the fact that many studies have found contrasting evidence that show that there are lower odds between paid work hours and poor SAH among men than women (21, 59).

Men and women spend time in short working hours for different reasons. It is well established that while majority of men spend time in short working hours for health reasons, majority of women spend time in short working hours to be able to combine paid work with childcare responsibilities (60, 61). Thus, the present results that short working hours in paid work are associated with less-than-good SAH among men but not women, may be because men try to meet their paid work responsibility regardless of their health conditions. Few studies have demonstrated that unlike women with poor SAH, men with poor SAH may involuntarily engage in precarious jobs with short working time, and this in turn, may have adverse health outcome (61). Furthermore, the association between long working hours and less-than-good SAH among men may be attributed to work overloads, and prolonged work stress which is linked to poor health status (58). Also, lack of statistical power due to lower frequency of women who engage in long working hours may most likely explain why we did not find a significant association in the relationship between long working hours and less-than-good SAH for women.

### Between-Country Differences of Work Hours and SAH

The multilevel logistic regression indicated that the association between paid work hours and less-than-good SAH for both men and women varied between countries in Europe, consistent with prior studies (8, 28). This was further corroborated in the multivariate analysis that was performed for each country in Europe. For example, we found the strongest association between working <31 hours and less-than-good SAH among workers in Romania (for women), Czech Republic (for men), Switzerland (for men), and Belgium (for women) respectively. On the other hand, the weakest association between working <31 hours and less-than-good SAH among workers was found in Denmark for both men and women. These findings may be explained by the differences in labor laws and regulations that exists in these countries (28, 62, 63). For instance, while there are strong labor regulation and labor unions in Denmark which may help improve the quality of working conditions for workers, the weak labor unions in Romania, Czech Republic, Switzerland, and Belgium (24, 64) may allow managers to have a strict control over the job process and thereby increase work-related stress (65). It is also possible that the significant association between short working hours and less-than-good SAH that were identified in these countries may be due to healthy worker effect (57).

In regards to the long working hours, we found the strongest association between working 50+ h and less-than-good SAH in Portugal (for men), Romania (for women), and Belgium (women) respectively. Nevertheless, there were no association between working 50+ h a week and less-than-good SAH for most of the countries in Europe including Netherland, Germany, Italy, Austria, Norway, Sweden, Luxembourg, France, and Spain. The present findings may partly be attributed to cross-country variation in work time regulation in terms of contractual work hours and overtime hours that allows workers in some countries to work longer hours than others (8, 26, 28, 63). For instance, our study revealed that while the average weekly working hours for Netherland, Germany, Italy, Austria, Norway, Sweden, Luxembourg, France, Spain, Denmark, and most of the countries in Europe are below 40 h for both men and women, the average weekly working hours for Czech Republic (for men) and Romania (for both men and women) are above 40 h. However, this reason does not explain the strong association among working women in Belgium since women reported averagely lower level of working hours.

Given the results of previous studies conducted on between-country differences in the relationship between work-life and health (66, 67), and in view of differences in welfare policies between countries in Europe, it is noteworthy that we expected to observe substantial differences in the relationship between working hours and less-than-good SAH between the countries in Europe. However, our findings suggest only small between-country variations (men: MOR = 1.63 vs. women: MOR = 1.74). It is possible that using countries as the macro level in the present analyses may explain the small variations that were observed (68). The percentage change in variance in the relationship between paid work hours and less-than-good SAH was slightly higher for women than men in Europe (men: MOR = 7.41% vs. women: MOR = 9.25%). This may be explained by the cross-country differences in social policies such as paid parental leave, and child and elderly care which helps men and women to combine both paid work and unpaid work (28, 62, 63). Unlike countries like Sweden, Norway, Finland, and Denmark that are characterized by dual-earner model and have generous and encompassing paid parental leave, and child and elderly care, countries like Belgium, Switzerland, Ireland, Portugal, Czech Republic, and Romania are characterized by the traditional breadwinner model and have minimal paid parental leave, and child and elderly care (24). Nonetheless, there is evidence that the dual-earner model is becoming more common across Europe leading to men and women participating in both paid and unpaid work (69). Besides, because women still spend more time in unpaid work compared to men, they may suffer from double burden role when combining paid and unpaid work (20), and this may subsequently have a negative association on their health as compared to men (45, 47). Also, economic crises and financial stress in some European countries has put a lot of pressure on married women to enter the labor market and accept jobs with low bargaining power and poor working conditions (70). Meanwhile, jobs with low bargaining power and poor working conditions have been shown to have higher risk of poor health (71). Another possible reason for the gender difference in the cross-country variations in the relationship between working hours and less-than-good SAH may be due to the fact that women generally report poorer health status compared to men (72).

### Sleep Disturbance and SAH

Contrary to another study (38), the present findings showed that sleep disturbance did not have a moderating effect in the association between paid work hours and less-than-good SAH for both men and women in Europe. Therefore, sleep disturbance may not be a key contributing factor to the relationship between paid work hours and less-than-good SAH in Europe. Nevertheless, the present findings should be taken with caution because while the study conducted by Nakata (38) conceptualized sleep status as a distinct factor of sleep duration, the current study conceptualized sleep status as a distinct factor of sleep disturbance. However, and despite the fact that those with shorter or longer sleep duration are most likely to report sleep disturbance, there is a conceptual distinction between sleep duration and sleep disturbance (73). Furthermore, unlike the study from Nakata (38) that was based on working adults in Asia (Japan), our study was based on working adults in Europe. Meanwhile the sleep status of those living in Asia and Europe may differ because of the difference in life style, behavior, socio-economic positions, and living environment (74). It is possible that the insignificant moderating role of sleep disturbance in the relationship between working hours and less-than-good SAH that was observed in the present studies may be due to the low level of sleep disturbance among working men and women in Europe (men = 2.36 ± 1.25 vs. Women = 2.62 ± 1.29).

### Policy Implications

The present study provides evidence for organizations, governments, policy makers, and researchers about gender differences and cross-country variations in the relationship between paid work hours and less-than-good SAH among workers in Europe. Governments and organizations must recognize the need and the importance of maintaining the health and well-being of workers. Promoting and maintaining health and well-being of workers is an avenue for increasing productivity and economic development for the nations. Therefore, governments through its agencies and actors must properly enforce the European Union Work time directive that limits standard work time to 48 h. This will to some extent, reduce the voluntary and involuntary engagement in long working hours in Europe. For instance, implementation of strict regulation of not working more than 50 h a week could possibly improve the general health and well-being of workers. In addition, there must be conscientious and continuous effort by governments and organizations to promote gender equity of paid work in Europe. This could be done by redesigning, developing, and implementing more generous work-family policies such as child and elderly care, and parental leave that allow men and women to combine working life with other aspects of life effectively.

### Limitations and Strengths

This study may be subject to several limitations. First, causality could not be established because of the cross-sectional nature of the study design (75). Indeed, there could be a possibility of reverse causality where less-than-good SAH may contribute to the observed short working hours. However, the possibility of a reverse causality where less-than-good SAH may lead to long working hours is low since workers who experience less-than-good SAH may rather reduce their working hours. Thus, to establish causality between working hours and SAH, future studies should adapt a longitudinal study design. Second, the present study could not adjust for behavioral and biological factors because they were not included in the EWCS 2015 data. In spite of this, socioeconomic factors such as education, and occupation that were adjusted for in this study are known to be important social determinants of health (58). Third, we used a cross-country approach to analyse the variations between countries in Europe. However, some studies have indicated that using a cross-country approach may not depict the true variation between the different countries (68). Therefore, and as recommended by Bambra et al. (68), future studies should use different approaches focusing on gross domestic product, legislation, social expenditure, demand for labor, and social policy indicators when analyzing the variations in the association between work hours and SAH in Europe. Fourth, the present study relied on subjective measures to assess health status. Meanwhile, subjective measure may have heterogeneity problems considering the fact that people with different socio-economic positions, demographic status, locations, cultural norms and values may employ different threshold to assess their health status (76). We suggest that similar studies in the future should use objective data in measuring the health status of workers in Europe.

Despite the above limitations, to our knowledge, this is the first study to investigate the relationship between paid work hours, sleep disturbance, and SAH with a contemporary comprehensive and nationally representative cross-country sample in Europe.

## Conclusions

In conclusion, the current study demonstrates that in Europe, there are gender differences and between-country variations in the association between time spent in paid work and less-than-good SAH, primarily because of the gender division of labor, and differences in social benefits, work time regulations, and labor laws and regulations that exist across countries. Although, the current study implies that sleep disturbance did not play a significant moderating role, sleep is an important factor in promoting good health. Further studies are required to investigate the concrete mechanisms and path ways through which time spent in paid work is linked to adverse health outcomes in Europe. Our study underscores the need for governments, organizations, policy makers, and employers to develop, and implement effective work time regulations, favorable work-family policies and labor regulations that promote gender equity in paid work hours, good working conditions and work environment for workers in Europe.

## Data Availability Statement

Publicly available datasets were analyzed in this study. This data can be found here: https://www.eurofound.europa.eu/surveys/european-working-conditions-surveys/sixth-european-working-conditions-survey-2015.

## Author Contributions

AM prepared the data, performed the statistical analyses, and drafted the manuscript. All authors have contributed to the conception and design of the study, interpretation of the data, revising the manuscript, and read and approved the final manuscript.

## Conflict of Interest

The authors declare that the research was conducted in the absence of any commercial or financial relationships that could be construed as a potential conflict of interest.

## Publisher's Note

All claims expressed in this article are solely those of the authors and do not necessarily represent those of their affiliated organizations, or those of the publisher, the editors and the reviewers. Any product that may be evaluated in this article, or claim that may be made by its manufacturer, is not guaranteed or endorsed by the publisher.
