# Supplementary material for: Working Hours, Sleep Disturbance and Self-Assessed Health in Men and Women: A Multilevel Analysis of 30 Countries in Europe
Source: Front Public Health. 2022 Apr 5;10:818359. doi: 10.3389/fpubh.2022.818359 (PMC9018983; doi:10.3389/fpubh.2022.818359)
Supplement: Supplementary file 1 [file Data_Sheet_1.pdf]

Appendix

Table A1: Bivariate relationship between self-assessed health and other variables by gender

Variable	Self-assessed Health			
	Men		Women	
Shift work	(0.067)	*	(0.000)	***
Education	(0.000)	***	(0.000)	***
Occupation	(0.000)	***	(0.000)	***
Living with a child	(0.016)	**	(0.000)	***
Marital status	(0.076)	*	(0.015)	**
Control	(0.000)	***	(0.001)	***
Sector	(0.037)	**	(0.014)	**
Type of Industry	(0.000)	***	(0.000)	***
Type of employment	(0.003)	***	(0.163)	NS
Working arrangement	(0.021)	**	(0.028)	**
Weekly hours	(0.000)	***	(0.000)	***
Age	0.213^{PB}	***	0.206^{PB}	***
Sleep Disturbance	-0.346^{RB}	***	-0.403^{RB}	***
Household size	-0.054^{PB}	***	-0.058^{PB}	***

Notes: Significance level:$*** \ p < 0.001$, $** < 0.05$, $* < 0.10$
(): is the Pearson Chi-Square
PB: is the Point Biserial correlation coefficient
RB: is the Rank Biserial correlation coefficient
NS: not significant

Table A2: Variance Inflation Factor (VIF) of independent and covariate variables among 14,603 working men from 30 countries of the EWCS 2015

Variable	VIF
Child	2.16
Household Size	2.03
Working Arrangement	1.84
Employment type	1.66
Occupation	1.57
Partner	1.54
Education	1.45
Age	1.23
Control	1.21
NACE	1.16
Shift work	1.1
Weekly hour	1.08
Sector	1.05
Sleep	1.01
Mean VIF	1.44

Table A3 Variance Inflation Factor (VIF) of independent and covariate variables among 15,486 working women from 30 countries of the EWCS 2015

Variable	VIF
Household size	2.01
Child	1.71
Working arrangement	1.48
Education	1.45
Occupation	1.43
Employment type	1.42
Partner	1.23
Age	1.15
Control	1.12
Shift work	1.07
Sector	1.07
Weekly hour	1.06
Type of industry	1.04
Sleep	1.02
Mean VIF	1.3

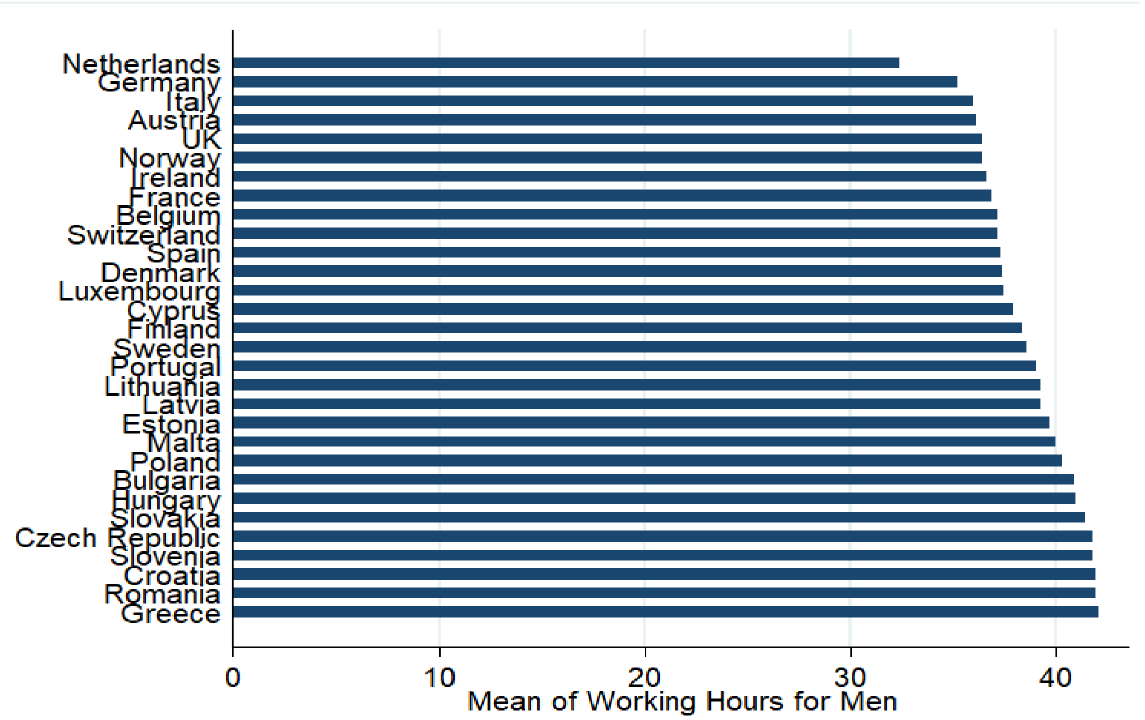

Fig A1 Average weekly working hours for working men in the 2015 European Working Condition Survey.

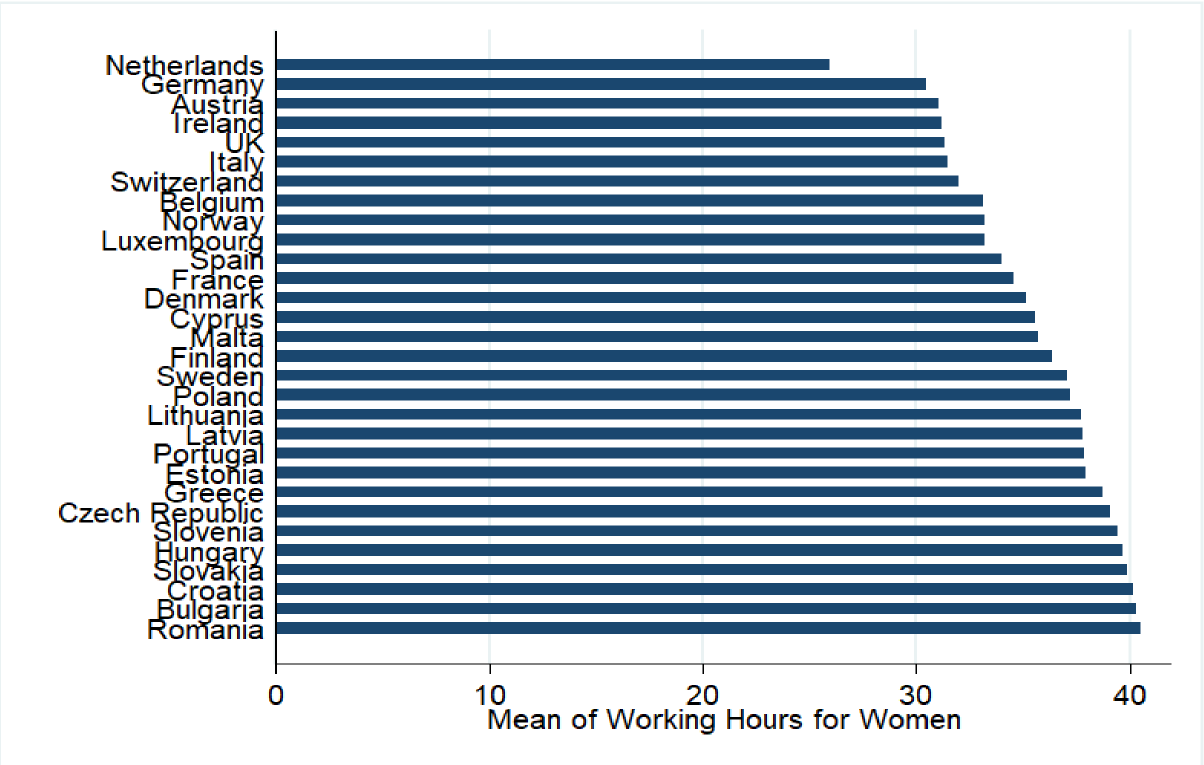

Fig A2 Average weekly working hours for working women in the 2015 European Working Condition Survey.

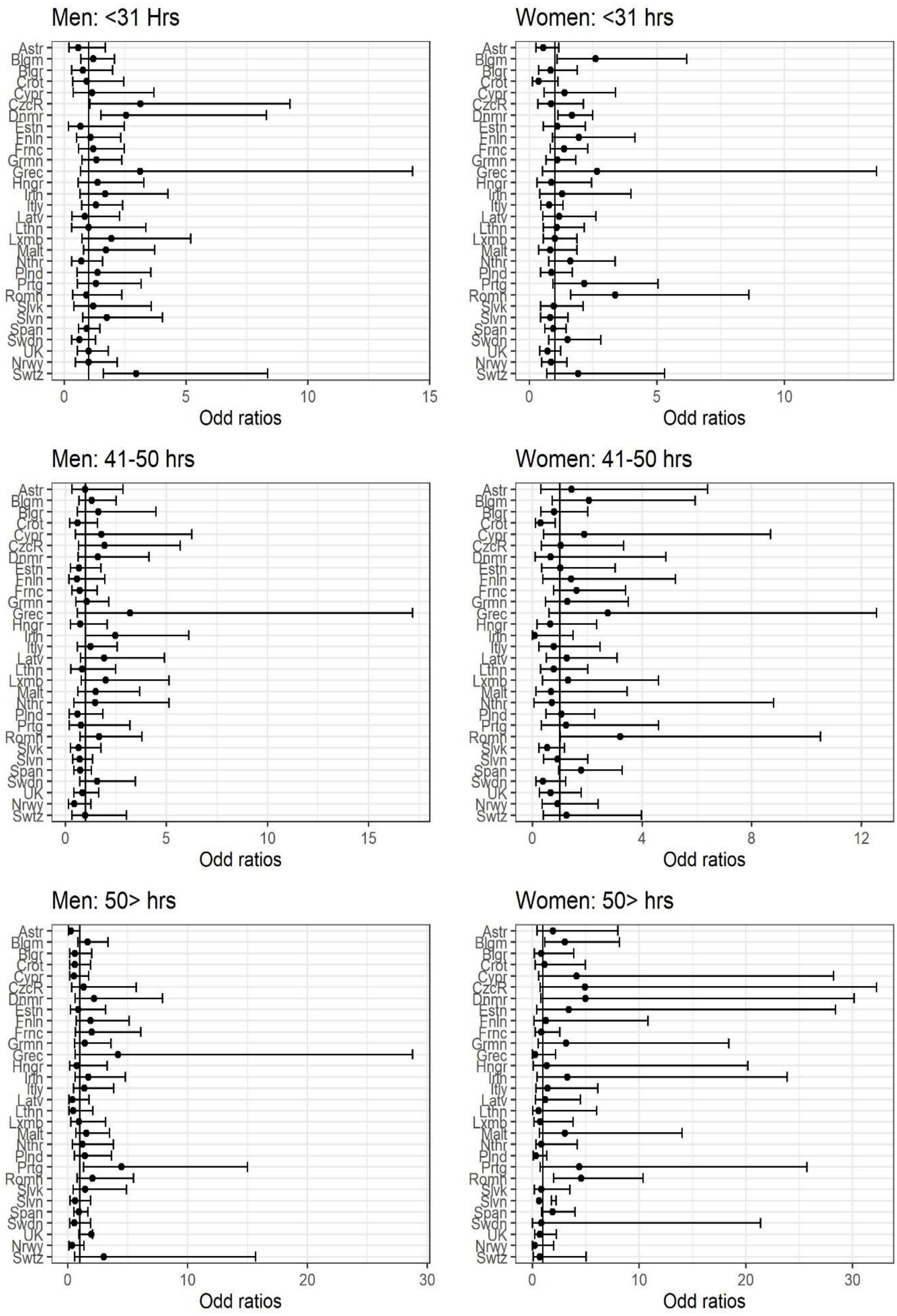

Fig A3 The association between paid work hours and self-reported health among men and women for 30 countries of the EWCS 2015
